# The immunosuppressive effects and mechanisms of loureirin B on collagen-induced arthritis in rats

**DOI:** 10.3389/fimmu.2023.1094649

**Published:** 2023-04-24

**Authors:** Yan Zou, Qianru Zhao, Xu Zhang, Hui Yu, Yongsheng Zhou, Ziyi Li, Min Xiao, Qiu Xiang, Lirong Zhang, Wenyi Shi, Haobo Tao, Lvyi Chen, Bing Han, Shijin Yin

**Affiliations:** ^1^ Department of Chemical Biology, School of Pharmaceutical Sciences, South-Central Minzu University, Wuhan, China; ^2^ Ethnopharmacology Level 3 Laboratory, National Administration of Traditional Chinese Medicine, Wuhan, China; ^3^ Department of Cardiology, Xuzhou Central Hospital, Xuzhou, Jiangsu, China

**Keywords:** loureirin B, Kv1.3 channel, immunosuppression, rheumatoid arthritis, calcium signalling pathway

## Abstract

**Introduction:**

Rheumatoid arthritis (RA) is a common disease mainly affecting joints of the hands and wrists. The discovery of autoantibodies in the serum of patients revealed that RA belonged to the autoimmune diseases and laid a theoretical basis for its immunosuppressive therapy. The pathogenesis of autoimmune diseases mainly involves abnormal activation and proliferation of effector memory T cells, which is closely related to the elevated expression of Kv1.3, a voltage-gated potassium (Kv) channel on the effector memory T cell membrane. Drugs blocking the Kv1.3 channel showed a strong protective effect in RA model animals, suggesting that Kv1.3 is a target for the discovery of specific RA immunosuppressive drugs.

**Methods:**

In the present study, we synthesized LrB and studied the effects of LrB on collagen- induced arthritis (CIA) in rats. The clinical score, paw volume and joint morphology of CIA model rats were compared. The percentage of CD3+, CD4+ and CD8+ T cells in rat peripheral blood mononuclear and spleen were analyzed with flow cytometry. The concentrations of inflammatory cytokines interleukin (IL)-1b, IL-2, IL-4, IL-6, IL-10 and IL-17 in the serum of CIA rats were analyzed with enzyme-linked immunosorbent assay. The IL-1b and IL-6 expression in joints and the Kv1.3 expression in peripheral blood mononuclear cells (PBMCs) were quantified by qPCR. To further study the mechanisms of immunosuppressive effects of LrB, western blot and immunofluorescence were utilized to study the expression of Kv1.3 and Nuclear Factor of Activated T Cells 1 (NFAT1) in two cell models - Jurkat T cell line and extracted PBMCs.

**Results:**

LrB effectively reduced the clinical score and relieved joint swelling. LrB could also decrease the percentage of CD4+ T cells, while increase the percentage of CD8+ T cells in peripheral blood mononuclear and spleen of rats with CIA. The concentrations of inflammatory cytokines interleukin (IL)-1b, IL-2, IL-6, IL-10 and IL-17 in the serum of CIA rats were significantly reduced by LrB. The results of qPCR showed that Kv1.3 mRNA in the PBMCs of CIA rats was significantly higher than that of the control and significantly decreased in the LrB treatment groups. In addition, we confirmed in cell models that LrB significantly decreased Kv1.3 protein on the cell membrane and inhibited the activation of Nuclear Factor of Activated T Cells 1 (NFAT1) with immune stimulus.

**Conclusion:**

In summary, this study revealed that LrB could block NFAT1 activation and reduce Kv1.3 expression in activated T cells, thus inhibiting the proliferation of lymphocytes and the release of inflammatory cytokines, thereby effectively weakening the autoimmune responses in CIA rats. The effects of immunosuppression due to LrB revealed its potential medicinal value in the treatment of RA.

## Introduction

Loureirin B (LrB) is a Resina Draconis (RD)-derived flavonoid and a traditional Chinese medicine with multifaceted effects on numerous diseases (1). LrB alone or combined with other RD constituents inhibited voltage-gated sodium channels, transient receptor potential vanilloid 1 channels, and acid-sensing ion channels in dorsal root ganglion (DRG) neurons and ameliorated inflammatory pain (2–4). Besides their analgesic effect, LrB and RD also possess promising immunosuppressive effects. Ethylacetated RD was found to inhibit inflammatory responses in vascular smooth muscle cells and macrophages by suppressing reactive oxygen species production in these cells (5). It has also been reported that LrB could inhibit the calcium ion (Ca^2+^) oscillations and nuclear factor of activated T cells c1 (NFATc1) activation in bone marrow macrophages of the Receptor activator of NF-κB ligand (RANKL)-induced osteoclastogenesis and ovariectomized osteoporosis model (6). A recent study showed that LrB could reduce the severity of inflammation in Crohn’s disease colon by inhibiting the expression levels of inflammatory cytokines interleukin-1 (IL-1), IL-6 and tumor necrosis factor-alpha (TNF-α) (7). However, the effects of LrB on rheumatoid arthritis (RA) and the mechanisms behind LrB-induced immune suppression have not been fully elucidated.

Rheumatoid arthritis (RA) is a chronic disease mainly involving joints of the hands and wrists (8, 9). Its clinical manifestations are joint swelling and inflammation of the infected joints with persistent and recurrent lesions, and a high frequency of disability (8, 9). The discovery of autoantibodies in the serum of patients gradually revealed that RA belonged to the autoimmune diseases and laid a theoretical basis for its immunosuppressive therapy (10, 11). The immunosuppressive agents which could decrease the cytokine release or block the signalling pathways of lymphocytes activation are important direction for drug development to treat RA (8, 9). The pathogenesis of autoimmune diseases mainly involves in abnormal activation and proliferation of effector memory T (T_EM_) cells, which is closely related to the overexpression of Kv1.3—a voltage-gated potassium (Kv) channel (12, 13). Activation of Kv1.3 channels will lead to the hyperpolarization of T_EM_ cells, thus providing the driving force for Ca^2+^ influx and a sharp elevation of intracellular Ca^2+^ concentration (14, 15). Intracellular Ca^2+^ signalling could subsequently activate the transcription, synthesis and secretion of various inflammatory cytokines such as IL-1, aggravating the autoimmune responses (14, 15). Drugs blocking Kv1.3 channels showed a strong therapeutic effect in RA model animals, suggesting that Kv1.3 is a new target for the discovery of specific RA immunosuppressive drugs (16, 17).

Our previous study found that LrB could inhibit the Kv1.3 channel, Ca^2+^ influx, and IL-2 secretion upon activation of Jurkat T cells (18). These results indicate that LrB may act as an immunosuppressive agent to treat or prevent autoimmune diseases such as RA. In this study, we applied LrB in a collagen-induced arthritis (CIA) rat model and found that LrB effectively reduced the clinical score and relieved joint swelling. The results of flow cytometry indicated that LrB decreased CD4+ and increased CD8+ T cells in peripheral blood mononuclear cells (PBMCs) and spleen cells in rats with CIA. An enzyme-linked immunosorbent assay (ELISA) showed that LrB could significantly reduce the concentration of IL-1β, IL-6 and other inflammatory cytokines in the serum of CIA rats. RT-PCR and Western Blot showed that the expression of Kv1.3 in the PBMCs of CIA rats was significantly higher than that of the control and significantly decreased in the LrB treatment groups. In addition, we confirmed in the cell models that LrB significantly decreased the expression of Kv1.3 and inhibited the activation of nuclear factor NFAT1. In summary, this study revealed that LrB could reduce Kv1.3 expression in activated T cells, thus inhibiting the proliferation of activated T cells and the release of inflammatory cytokines, thereby effectively weakening the autoimmune responses in CIA rats. The effects of immunosuppression due to LrB revealed its potential medicinal value in the treatment of RA.

## Materials and methods

### Chemicals

**Figure 1:**
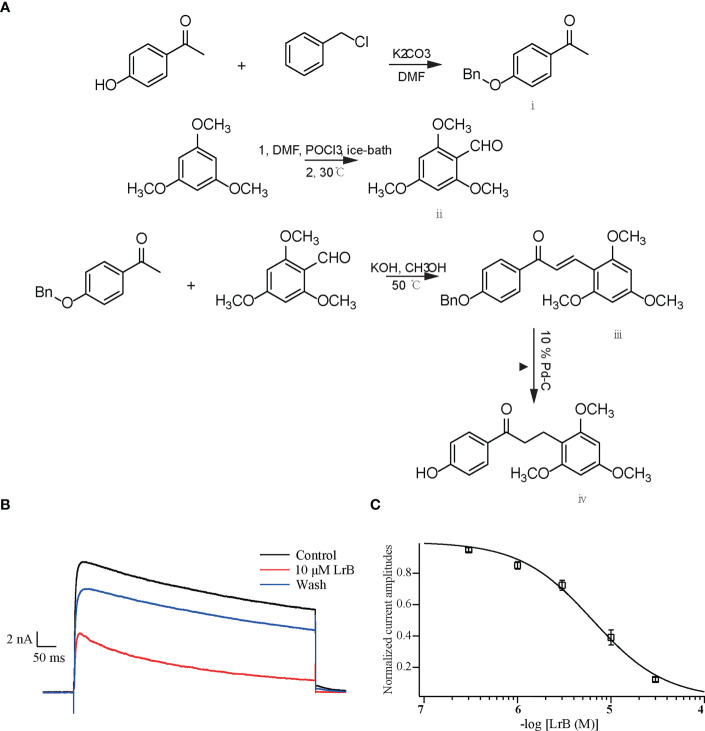
Synthetic LrB inhibited Kv1.3 currents. **(A)** Synthetic pathway of LrB. **(B)** Representative traces of Kv1.3 current in HEK293T cells in the Control, 10 μM LrB and Wash treatments. **(C)** Concentration-response curve of LrB inhibition of the Kv1.3 channel (n = 5, mean ± SEM).

LrB and its derivatives were synthesized by our group (**Figure 1A**) and lyophilized into power. Dimethyl sulfoxide was bought from Panreac AppliChem (Darmstadt, Germany), DMEM High-Glucose, 1640 medium, fetal bovine serum (FBS), penicillin-streptomycin solution, 0.25% trypsin solution and Opti-MEM were ordered from Gibco (New York, NY, USA), 100 μM poly-L-lysine solution from Sangon Co., Ltd (Shanghai, China), and lipofectamine 2000 from Invitrogen (Carlsbad, CA, USA). Bovine type II collagen was bought from Chondrex (WA, USA), incomplete Freund’s adjuvant (IFA) from Sigma (St. Louis, MO, USA), Dexamethasone from Xianju Pharmaceutical Co., Ltd. (Hangzhou, Zhejiang, China), Percoll separation medium from GE Healthcare (Waukesha, WI, USA) and red blood cell lysate from Biosharp Life Sciences (Hefei, Anhui, China). Phytohemagglutinin (PHA) and Concanavalin A (ConA) were bought from Yeasen Company (Nanjing, Jiangsu, China). LrB dry powders were dissolved in dimethyl sulfoxide to prepare 100 mM stock solutions, and external solutions were used to prepare different concentrations.

### HEK293T cell culture and transfection

HEK293T cells were cultured in DMEM High-Glucose with 10% FBS and 1% penicillin-streptomycin. The plasmid pIRES2-EGFP-mKv1.3 (gifted from Professor Wenxin Li laboratory, School of Life Sciences, Wuhan University, Wuhan, China) was applied to transfect the cells to express mouse Kv1.3 channels (18). The plasmid and transfection reagent lipofectamine 2000 were diluted in Opti-MEM, respectively. After 5 min, the two were mixed and left to stand for 20 min at room temperature. The mixture was then added to HEK293T cells preseeded in a 24-well cell culture plate, and replaced with complete medium after 5 h. Electrophysiological experiments were performed 24–48 h after cell plating when the transfection efficiency of plasmid pIRES2-EGFP-mKv1.3 reached 70-80%.

### Electrophysiology experiment

The extracellular solution used to record the Kv1.3 current contained: 5 mM KCl, 140 mM NaCl, 10 mM HEPES, 2 mM CaCl_2_, 1 mM MgCl_2_ and 10 mM D-Glucose (pH 7.4 with NaOH). The internal solution contained: 140 mM KCl, 1 mM MgCl_2_, 1 mM EGTA, 3 mM Na_2_ATP, and 10 mM HEPES (pH 7.2 with KOH). The whole-cell voltage clamp was recorded using an EPC9/2 amplifier, and the experimental temperature was 20–25°C. The resistance of the microelectrode glass capillary was around 2–5 MΩ. The cells were recorded under a whole-cell mode, the membrane potential of the cells was clamped at −60 mV, and a 400 ms step, +50 mV depolarizing current stimulation was provided every 20 s to activate the Kv1.3 channel currents exogenously expressed in HEK293T cells.

### CIA rat model

Wistar rats were bought from Wuhan Center for Disease Control and Prevention. All animal experiments were conducted according to the rules of the National Institutes of Health Guide for the Care and Use of Laboratory Animals and strictly followed the guidelines of the Institutional Animal Care and Use Committees (IACUC). The IACUC checked all protocols and approved this study, and the animal ethical approval number is 2020-scuec-028. One volume of IFA was mixed with an equal volume of collagen solution (2 mg/mL in 0.05 M acetic acid) and stirred at low speed (1000–3000 rpm) with a hand-held homogenizer (19). The solution was placed in an ice-water bath to keep the emulsion cool during mixing. Animals were divided into the control group and immunized group. Animals in the immunized group were inoculated with immunized antigens and the sham group were inoculated with saline. 200 μL (200 mg collagen/rat) of the emulsion was injected subcutaneously at the base of the tail. To ensure a high incidence and severity of arthritis, a booster injection was given on day 7 after the primary immunization. Collagen-IFA emulsions were prepared as described above and 100 μL of the emulsions were administered subcutaneously in the tail (19). The incidence for RA was 90% and the 10% rats were left according to their clinical scores and volumes of hindlimbs. The successfully induced CIA rats were randomly divided into the model group, LrB treatment group, and Dexamethasone (Dex) treatment group (positive control) (20). On the thirteenth day after the first immunization, the animals were intraperitoneally injected with 30 mg/kg body weight of LrB every day. The positive group was intraperitoneally injected with 5 mg/kg Dex, and the model group was intraperitoneally injected with 0.5 mL of saline every day.

### Clinical score and joint swelling measurement

CIA model rats generally begin to have clinical symptoms such as redness, swelling and pain 10–14 days after the injection of antigen emulsion (21). The degree of joint swelling in CIA rats is scored on a scale of 0 to 4, with 0 points: no arthritis; 1 point: redness and swelling of the little toe joint; 2 points: redness and swelling of the little toe joint and plantar; 3 points: swelling of the paws below the ankle joint; 4 points: the entire toe joint is swollen. A score of 1 or more was defined as RA. Arthritis scores (clinical scores) were summed for each joint lesion score in each rat. The swollen volume of the hindlimb foot was measured with a toe swelling instrument every three days.

### Morphological and immunological analysis

On the 28th day after the injection of antigen emulsion, the rats were anesthetized with isoflurane (RWD, Shenzhen, China) and sacrificed, and the degree of bone lesions in the joints was detected by micro-CT. The feet were excised, and the outer skin was peeled off, then placed in paraformaldehyde fixative solution for at least 48 h, and decalcified in EDTA solution for at least 4 weeks. After complete decalcification, the joints were embedded in paraffin blocks and sliced. The slices were stained with hematoxylin-eosin (HE), and the pathological changes in synovium and cartilage were observed by light microscopy. The histology images were scored according to the diseased arthritic infiltrates, synovial hyperplasia, cartilage damage and bone resorption in a double-blinded manner (22).

### PBMC extraction

The PBMCs from rats were separated with Percoll separation medium. The gradient Percoll separation solution was prepared as follows: Percoll: 10 × Phosphate-Buffered Saline (PBS): 1 × PBS = 6.3:0.7:3. The collected peritoneal venous blood from rats was diluted with 1 × PBS in an equal proportion on a sterile ultra-clean bench. 5 mL of Percoll separation solution was added to a 15 mL centrifuge tube. The diluted blood was spread on the upper layer of the separation solution in the centrifuge tube in equal proportions and centrifuged in a horizontal centrifuge at 1,500 g for 40 min at room temperature (23). After centrifugation, the lymphocytes aggregated in the middle layer of the blood plasma and the separation solution. A Pasteur pipette was inserted into the middle layer to carefully aspirate and transfer cells to another centrifuge tube and the cells were centrifuged for 5 min at 1,500 g. The cells were washed with 5 mL of 1 × PBS and then centrifuged at 1,500 g. The supernatant was discarded, and the cells were resuspended in 1640 complete medium. The cell density was counted with a Cellometer K2 dual fluorescence cell analyzer (Nexcelom, San Diego, CA, USA), and seeded in cell culture bottles.

### Spleen cell isolation

After sacrifice, the rat spleen was removed quickly and rinsed with 3 mL 1 × PBS with a L-shaped syringe. The spleen was ground gently with the flat end plunger of 5 mL syringe and the cells were collected in a Petri dish (24). The cell suspension was transferred to a sterile centrifuge tube and centrifuged with 1,500 rpm for 3 min. The supernatant was discarded, and the cells were resuspended in red blood cell lysate on ice for 3 min and centrifuged again. The cells were washed twice with 1× PBS and filtered with a cell strainer. The obtained spleen cell suspension was used for flow cytometry.

### Flow cytometry

Extracted PBMCs from the sham group or CIA model rats were incubated in APC-labelled rat CD3 antibody (eBioscience, San Diego, CA, USA), FITC-labelled rat CD4 antibody (eBioscience), and PE-labelled rat CD8 antibody (eBioscience) for 30 min at room temperature and sorted with FACS Calibur (BD Biosciences, Allschwil, Switzerland). Isolated spleen cells from the sham group or CIA model rats were incubated in PE-Cy7-labelled rat CD45 antibody (eBioscience), FITC-labelled rat CD3 antibody (eBioscience), APC-labelled rat CD4 antibody (eBioscience), and PE-labelled rat CD8 antibody (eBioscience) for 30 min at room temperature and sorted with FACS Calibur (**Figure S1**). For quantification of Kv1.3 expression on PBMC membrane, the extracted PBMCs were incubated with FITC-labelled rat Kv1.3 antibody (Cusabio, Wuhan, Hubei, China) and analyzed with FACS Calibur. For quantification of Kv1.3 expression in regulatory T cells, isolated spleen cells were stained with APC-labelled rat CD4 antibody (eBioscience), PE-labelled rat CD25 antibody (eBioscience) and FITC-labelled rat Kv1.3 antibody (Cusabio) for 30 min at room temperature. Then the cells were fixed and permeabilized with Foxp3/Transcription Factor Staining Buffer Set (eBioscience). After brief wash, the cells were incubated with PE-Cy5.5-labelled rat FoxP3 antibody (eBioscience) for 30 min at room temperature and sorted with FACS Calibur (**Figure S2**). The data were analyzed with BD FACS Diva software (BD Biosciences).

### ELISA

Jurkat T cells and PBMCs were seeded in 96-well cell culture plates at 5 × 10^4^ cells/well in a final volume of 200 μL. Cells in the control group were treated with 20 μL 1 × PBS instead of drug solution, and the treatment groups each received the same volume of 10 μM LrB solution, the cells were placed in a CO_2_ incubator and incubated with the drug for 60 min, then the stimulators PHA and ConA were added, and 1×PBS was added to the cells in the control group. The cells were cultured in the CO_2_ incubator for 24 h. The cell culture supernatant was collected by centrifugation for 5 min at 1,500 rpm. The level of inflammatory factors released was detected by ELISA according to the ELISA kit instructions (18). Rat IL-1β, IL-2, IL-4, IL-6, IL-10, IL-17 ELISA and Human IL-2 kits were bought from Sizhengbai (Beijing, China)

### qPCR

The cells were collected by centrifugation for 5 min at 1,500 rpm. The supernatant was discarded, and 1 mL PBS was added to wash the cells. Next, 1 mL of Trizol (Invitrogen) was added to every 5 × 10^6^ cells and the cells were pipetted repeatedly until no obvious particles existed. A 1/5 volume (200 μL/1 mL) of chloroform was added to Trizol to lyse the cells. The cells were lysed on ice for 5 min and centrifuged for 5 min at 12,000 rpm and 4°C. The upper aqueous layer was transferred to a new centrifuge tube. 600 μL of pre-cooled isopropanol was added and the mixture was vortexed and kept on ice for 10 min. The mixture was centrifuged for 10 min at 12,000 rpm and 4°C. The supernatant was discarded and 1 mL of 75% alcohol in DEPC water was added to suspend the precipitant. The mixture was centrifuged for 5 min at 12,000 rpm and 4°C and the supernatant was discarded. The product was dried with the lid open for 5 min. 30 μL of DEPC water was added to dissolve the RNA precipitate. RNA was reverse transcribed according to the RevertAid™ First Strand cDNA Synthesis Kit instructions (K1622, Thermo Scientific). Then qPCR was conducted according to the quantitative PCR kit instructions (A25741, Thermo Scientific). The primers for qPCR were as follows:

Human(h)β-actin -FP: 5’-CACGATGGAGGGGCCGGACTCATC-3’

hβ-actin-RP: 5’-TAAAGACCTCTATGCCAACACAGT-3’

h-Kv1.3-FP: 5’-ACGCTGTGCATCATCTGGTT-3’

h-Kv1.3-RP: 5’-TGCTGAAACCTGAAGTGGGG-3’

Rat(r)β-actin-FP: 5’- GAACCCTAAGGCCAACCGTGA-3’

rβ-actin-RP: 5’-CTTGCTCGAAGTCTAGGGCA-3’

rIL-1β-FP: 5’-CTTGTTTCATTCTGAGCCTCCTC-3’

rIL-1β-RP: 5’-ATATGTCGGGCTGGTTCCAC-3’

rIL-6-FP: 5’-GATTGTATGAACAGCGATGATGC-3’

rIL-6-RP: 5’-AGAAACGGAACTCCAGAAGACC-3’

riNOS-FP: 5’-GGATATCTTCGGTGCGGTCTT-3’

riNOS-RP: 5’-CTGTAACTCTTCTGGGTGTCAGA-3’

rCD86-FP: 5’-ACAGCAAAAGACACCCACGG-3’

rCD86-RP: 5’-CTTGTTTCATTCTGAGCCTCCTC-3’

rCD206-FP: 5’-TGTTTTGGCTGGGACTGACCTA-3’

rCD206-RP: 5’-CGGGTGTAGGCTCGGGTAGTAG-3’

rKv1.3-FP: 5’-GACCCTTCTTCGGGTTT-3’

rKv1.3-RP: 5’-CAGTGGAGTTGCCCGTTTTG-3’

### Western blot

Cultured cells were collected by centrifugation for 5 min at 6,000 rpm and washed with ice cold 1 × PBS twice. The cells were lysed with Radio Immunoprecipitation Assay solution (Beyotime, Peking, China) for 30 min. The lysis solution was centrifuged again for 15 min at 12,000 rpm and 4°C and the supernatant was collected and mixed with 2 × Sodium dodecyl sulfate (SDS) loading buffer and boiled for 5 min. Before loading, the total protein concentration in each group was determined by the BCA kit and scanned with a microplate photospectrometer (SPARK 10M, Tecan, Hombrechtikon, Switzerland). Proteins were loaded based on the total protein concentration of each group to ensure equal quantities per lane. Proteins were separated on a 10% SDS-polyacrylamide gel and transferred to polyvinylidene fluoride (PVDF) membranes (Millipore, Billerica, MA, USA). The membranes were blocked with 10% nonfat milk and incubated overnight at 4°C with the following antibodies: rabbit polyclonal antibody against extracellular part (485-575 amino acids) of Kv1.3 (1:500; Absin, Shanghai, China), rabbit monoclonal antibody against Na^+^-K^+^ ATPase α1 (1:1000; Beyotime, Shanghai, China), rabbit polyclonal antibody against NFAT1 (1:500; Affinity Biosciences, Nanjing, Jiangsu, China), and rabbit polyclonal antibody against Lamin B1 (1:1000; Beyotime). After washing in tris-buffered saline (TBS) with 0.3% Tween three times for 45 min, the membranes were incubated with HRP conjugated goat anti-rabbit IgG (1:10,000; Abclonal, Boston, MA, USA) for 2 h at room temperature. Chemiluminescent signals were generated using a Super Signal West Pico trial kit (Pierce Protein Biology, Thermo Fisher Scientific, Waltham, MA, USA) and detected using the ChemiDoc XRS System (Bio-Rad, Hercules, CA, USA). Image Lab software (Bio-Rad, Hercules) was used for background subtraction and quantification of immunoblotting data.

### Immunofluorescence of Jurkat T cells

The Jurakt T cells pre-treated with stimulant and LrB were seeded on the 10 µg/mL poly-L-lysine coated coverslips. After incubated for 2 h, the cells were washed with 1× PBS and fixed with 4% paraformaldehyde for 20min. The cells were permeabilized with 1× PBS containing 0.1% TritonX-100 (PBST) for 15min. Immunostaining blocking solution (Beyotime, Shanghai, China) was applied for 30min and incubated with the Kv1.3-FITC antibody (Cusabio) at 4°C overnight. The samples were washed with 1× PBS wash 3 times, then the coverslips were mounted on slides with anti-fluorescent quenching agent (containing DAPI). The images of immunofluorescence were captured with 63 × Confocal microscope (LSM900, Zeiss, Germany).

### Statistical analysis

All experimental data are expressed as mean ± sample standard error (mean ± SEM), where n represents the number of independent experiments. Differences between groups were statistically analyzed by the t-test or one-way ANOVA, and a value of P ≤ 0.05 was considered statistically significant. The current data collected by the amplifier was analyzed and calculated by Pulse, and the data were fitted using Igor Pro 4 software (WaveMetrics, Lake Oswego, OR, USA) to the concentration-response curve. Using Igor Pro 4 software, concentration–response curves were fitted according to the following modified Hill equation:


$${\text{I}}_{\text{toxin}}={\text{I}}_{\text{control}}=1/1+([\text{peptide}]/\text{I}{\text{C}}_{50}),$$

where I represents the peak current; [peptide] represents the concentration of drug.

The IC_50_ was obtained by four-parametric nonlinear regression analysis constraining bottom to 0 and top to 1.

## Results

### Effects of synthetic LrB on Kv1.3 currents and clinical score in CIA model rats

The plasmid containing the gene encoding the mKv1.3 channel was transfected into HEK293T cells. After 24 h, the cells were resuspended and spread on a coverslip. The cells were clamped at −60 mV and depolarized with +50 mV step for 400 ms every 20 s to activate the Kv1.3 channel current. When the current was stable, the elution system with 1.0 mL/min flow rate was placed close to the cells to start drug delivery, and different concentrations of LrB solutions were given. The results showed that LrB effectively inhibited the Kv1.3 current, 30 μM LrB almost completely inhibited the mKv1.3 channel current exogenously expressed on HEK293T cells, and this inhibitory effect was partially reversed by elution with extracellular solution (**Figure 1B**). The statistical results of the experimental data showed that the inhibitory effects of LrB on Kv1.3 channel currents were concentration dependent. The concentration-response curve of Kv1.3 was fitted to the Hill equation, and the IC50 value of LrB acting on the mKv1.3 channel was 6.33 ± 0.49 μM (**Figure 1C**).

**Figure 2:**
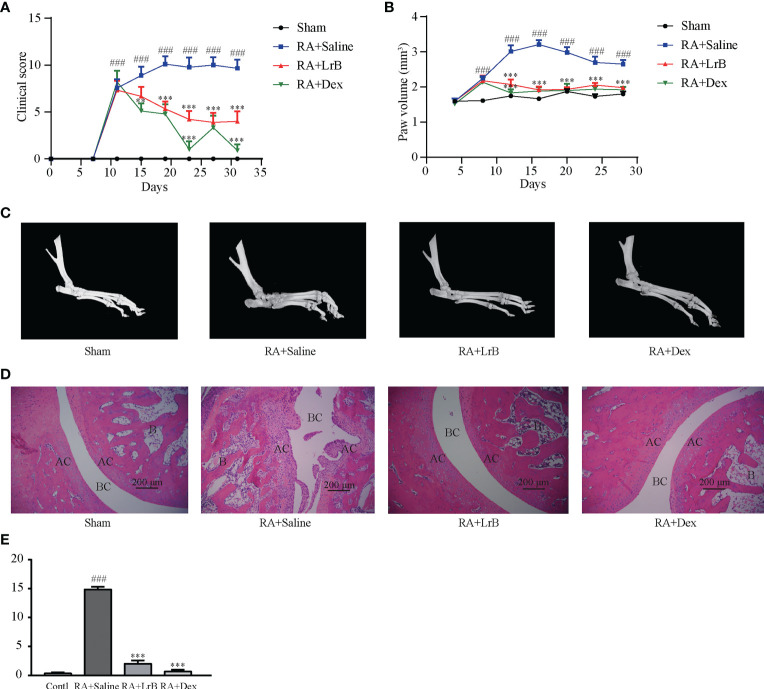
LrB alleviated the progression of rheumatoid arthritis. **(A)** Diagram of the average clinical scores in CIA rats. The degree of joint swelling in CIA rats is scored on a scale of 0 to 4, with 0 points: no arthritis; 1 point: redness and swelling of the little toe joint; 2 points: redness and swelling of the little toe joint and plantar; 3 points: swelling of the paws below the ankle joint; 4 points: the entire toe joint is swollen. **(B)** Diagram of the average toe swelling volume (mm^3^) in CIA rats. **(C)** The hind feet of rats scanned with micro-CT. The bones and joints of the rats in the control, LrB and Dex treatment group were smooth and no bone hyperplasia or osteoporosis were observed, CIA model group showed rough articular surfaces, thickened bones, and severe bone erosion. **(D)** HE staining of rat joint sections, the abbreviations in the images are Bone (B), articular cartilage (AC), and bone cavity (BC). The articular cartilage structure of the rats in the control, LrB and Dex groups were clear, the synovium showed no hyperplasia or bone erosion, and there was no inflammatory cell infiltration in the tissue. The synovial membrane of the rats in the CIA model group showed abnormal hyperplasia, villi-like protrusions, a large number of inflammatory cells infiltrating the joints, and severe cartilage and bone erosion. **(E)** Total histological score of CIA rats according to the diseased arthritic infiltrates, synovial hyperplasia, cartilage damage and bone resorption. (Results are expressed as the mean ± SEM, and tested with One Way ANOVA. ###, P ≤ 0.001, compared to the Sham group; * * P ≤ 0.01, * * * P ≤ 0.001, compared to the RA+Saline group).

The CIA rat model was successfully induced by the prepared IFA and the antigen of bovine type II collagen. The immunized rats started to develop symptoms such as redness and swelling of the hindlimbs and feet, and slight weakness of the hindlimbs from the 13th day (19, 21). After the peak period of onset, the joint redness and swelling improved slightly, but functional damage to bone and joint appeared (19, 21). The clinical scores in the LrB treatment group were significantly lower than those in the model group from day 15 of immunization (**Figure 2A**). The results of the toe swelling test showed that the degree of toe swelling in the CIA model group was significantly higher than that in the control group, and the degree of toe swelling in the CIA rats treated with LrB was effectively relieved (**Figure 2B**). These results indicated that LrB had therapeutic effects in the RA model rats.

### Effects of LrB on joint morphology of CIA rats

Joint bone hyperplasia and/or destruction is an important cause of joint stiffness and ultimately loss of joint function in RA patients (25). To test whether LrB can improve the degree of bone and joint lesions in CIA rats, we used micro-CT to scan the hind feet of rats. The CT scan results revealed that the bones and joints of the rats in the CIA model group showed rough articular surfaces, thickened bones, and severe bone erosion (**Figure 2C**). The bones and joints of the rats in the control group were smooth and no lesions were observed (**Figure 2C**). The articular surfaces of the LrB treatment group were relatively smooth, and there was no obvious bone hyperplasia and osteoporosis, indicating that LrB could effectively improve the symptoms of joint bone destruction in CIA rats.

Diseased arthritic infiltrates, synovial hyperplasia, synovial protrusion into the joint cavity, cartilage damage and erosion are important histological features in RA patients (25). The sections were stained with HE, and pathological damage in CIA rats was observed under the microscope. The articular cartilage structure of the rats in the control group was clear, the synovium showed no hyperplasia, the cartilage was intact, and there was no inflammatory cell infiltration in the tissue (**Figures 2D, E**). The synovial membrane of the rats in the CIA model group showed abnormal hyperplasia, villi-like protrusions, a large number of inflammatory cells infiltrating the joints, and severe cartilage erosion (**Figures 2D, E**). The structure of the articular cartilage in the LrB treatment group was clear, and there was no obvious hyperplasia or cartilage in the synovium (**Figures 2D, E**). The joint slice was relatively complete, with only a few inflammatory cells infiltrating the tissue. These results indicated that LrB could effectively improve the inflammatory symptoms and synovial hyperplasia of the diseased joints in CIA rats.

### Effects of LrB on polarization of T cells and cytokines in CIA rats

**Figure 3:**
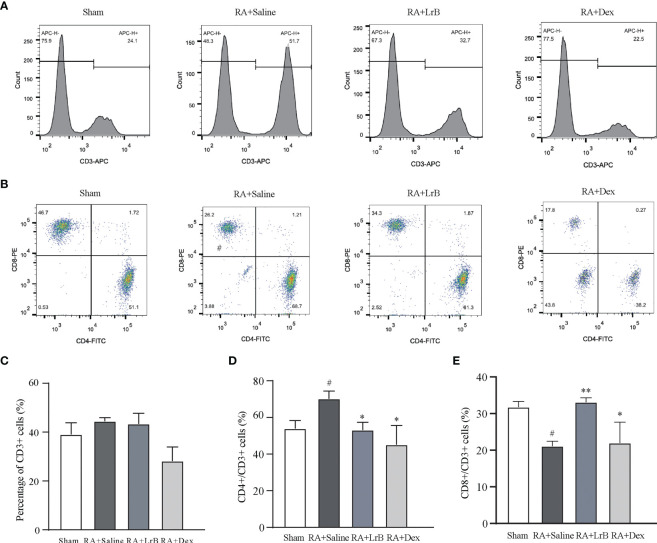
LrB changed the ratio of PBMCs in CIA rats. **(A)** Staining histogram of CD3+ T-cell subtype in peripheral blood mononuclear cells (PBMCs) of Sham and CIA rats with different treatments. **(B)** Scatter diagram of CD4+ and CD8+ T-cell subtypes in CD3+ T cells of CIA rats. **(C)** The proportion of CD3+ T cells in PBMCs of rats in each group. **(D)** The proportion of CD4+ T-cell subtype in CD3+ T cells. **(E)** The proportion of CD8+ T-cell subtype in CD3+ T cells. (Results are expressed as the mean ± SEM, and tested with One Way ANOVA. # P ≤ 0.05, compared to the Sham group; * P ≤ 0.05, ** P ≤ 0.01, compared to the RA+Saline group).

**Figure 4:**
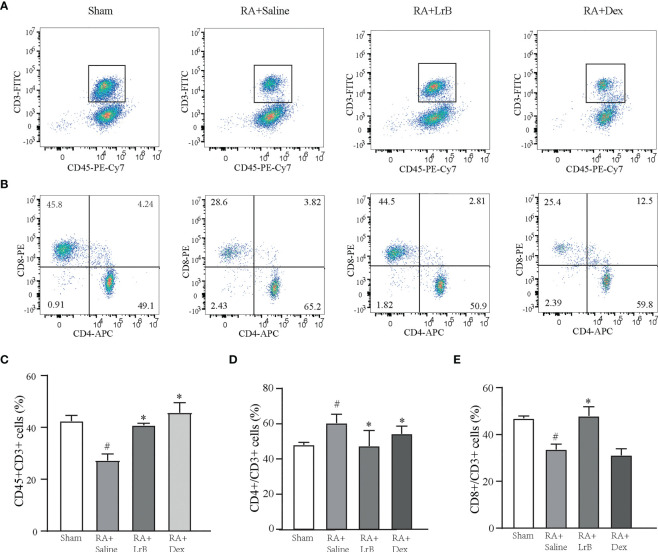
LrB changed the ratio of spleen cells in CIA rats. **(A)** Scatter diagram of CD45+CD3+ T-cell subtype in spleen cells of Sham and CIA rats with different treatments. **(B)** Scatter diagram of CD4+ and CD8+ T-cell subtypes in CD45+CD3+ T cells of CIA rats. **(D)** The proportion of CD4+ T-cell subtype in CD45+CD3+ T cells. **(E)** The proportion of CD8+ T-cell subtype in CD45+CD3+ T cells. (Results are expressed as the mean ± SEM, and tested with One Way ANOVA. # P ≤ 0.05, compared to the Sham group; * P ≤ 0.05, compared to the RA+Saline group). **(C)** The proportions of CD45+CD3+ T cells in total spleen cells.

Antigen-activated T cells selectively enter specific parts of the joint with the interaction of chemokine (CK) and its receptors, homing receptors, and addressins, causing slippage (26). Inflammatory infiltration of membranous tissue leads to exacerbation of the disease. Distributed T cells in lymphoid organs and lymphoid tissues generate immune responses to antigens carried by the blood and lymphatic circulation (26). The immune response produces pro-inflammatory cytokines that further exacerbate inflammatory infiltration and cartilage damage at diseased sites in RA patients. The abnormal proportion of T lymphocytes is one of the important indicators reflecting the condition of RA patients. To verify whether LrB inhibited abnormal proliferation of T lymphocytes in peripheral blood, we labelled T cells in peripheral blood with anti-rat CD3-APC, CD4-FITC and CD8-PE antibodies and detected the cells using flow cytometry. The results showed that the proportion of CD3+ T cells in the CIA model group were not different significantly with LrB treatment group but LrB decreased the elevated CD4+ T cells in CIA model group (**Figures 3A–D**). The proportion of CD8+ cells in the peripheral blood of the rats in the CIA model group was significantly lower than that in the peripheral blood of the rats in the control group (**Figures 3A, B, E**). We also labelled the rat spleen cells with anti-rat CD45-PE-Cy7, CD3-FITC, CD4-APC and CD8-PE antibodies and detected the cells using flow cytometry. The proportion of CD3+ and CD8+ T cells in the spleen of the rats in the CIA model group was significantly decreased, while CD4+ T cells was significantly increased (**Figures 4A–E**). These results show that LrB can effectively reverse the abnormal proliferation of T cells, thereby producing immunosuppressive effects.

**Figure 5:**
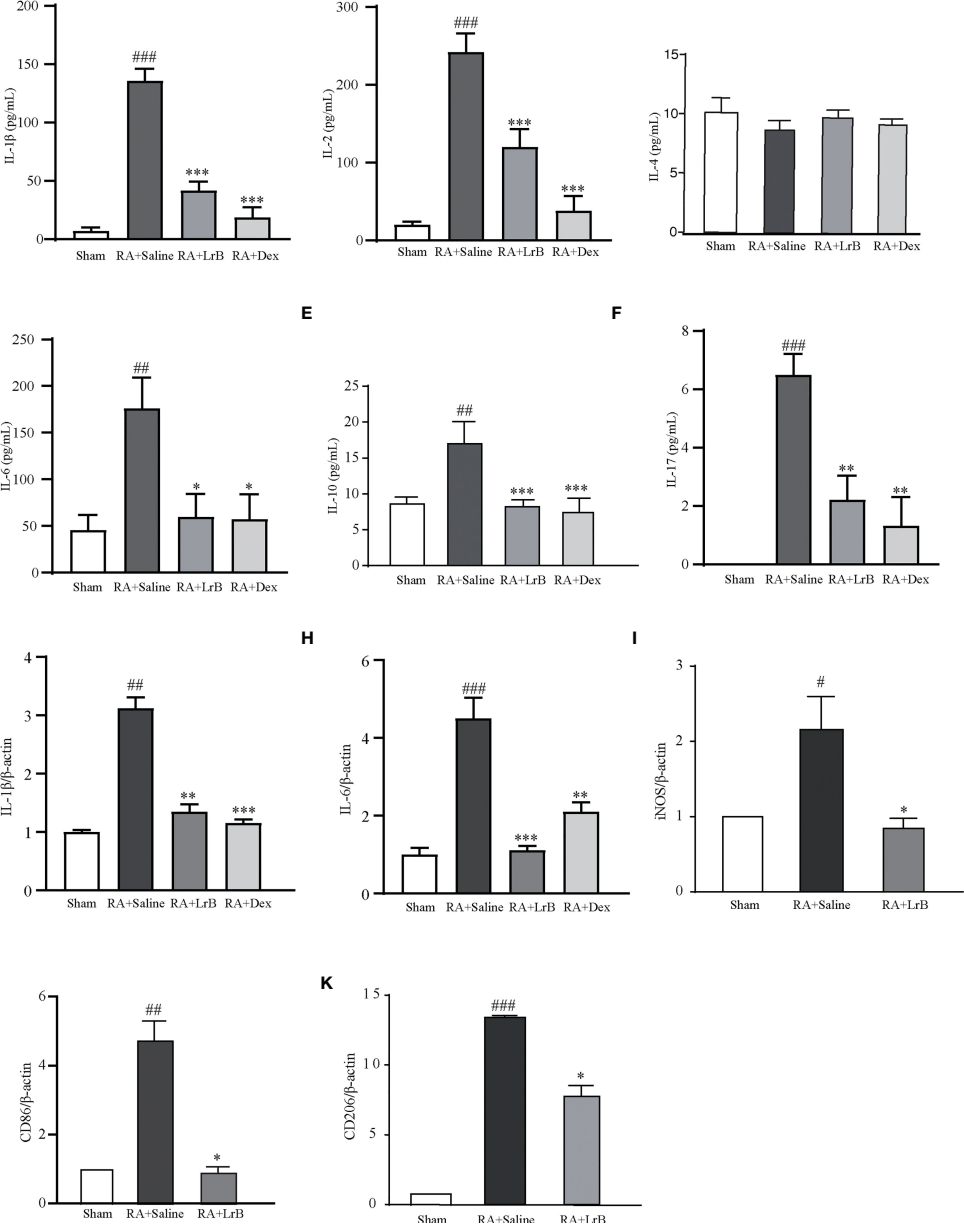
LrB decreased cytokine release and Kv1.3 expression in PBMCs from CIA rats. **(A)** The cytokine IL-1β concentration in the serum of CIA rats detected by ELISA (n=5). **(B)** The cytokine IL-2 concentration in the serum of CIA rats detected by ELISA (n=5). **(C)** The cytokine IL-4 concentration in the serum of CIA rats (n=5). **(D)** The cytokine IL-6 concentration in the serum of CIA rats (n=5). **(E)** The cytokine IL-10 concentration in the serum of CIA rats (n=5). **(F)** The cytokine IL-17concentration in the serum of CIA rats (n=5). **(G, H)** IL-1β **(G)** and IL-6 **(H)** mRNA in joint cartilage as shown by qPCR (n = 6). **(I–K)** iNOS **(I)**, CD86 **(J)**, CD206 **(K)** mRNA in spleen cells as shown by qPCR (n=4). (Results are expressed as the mean ± SEM, and tested with One Way ANOVA. #, P ≤ 0.05, ##, P ≤ 0.01, ###, P ≤ 0.001, compared to the Sham group; * P ≤ 0.05, * * P ≤ 0.01, * * * P ≤ 0.001, compared to the RA+Saline group).

The cytokines IL-1β, IL-2, IL-4, IL-6, IL-10 and IL-17 are involved in the development of RA (26, 27). Studies have found that the levels of cytokines in the serum of RA patients are significantly increased, and their levels are positively correlated with severity of the disease in RA patients (27). Reducing the secretion of cellular inflammatory factors may be a new strategy for the treatment of RA (26, 27). We tested whether LrB could down-regulate the levels of these cytokines in serum of CIA rats using ELISA. Compared with the control group, the levels of IL-1β, IL-2, IL-6, IL-10 and IL-17 in the serum of rats in the CIA model group were significantly increased, the level of IL-4 did not show a significant difference between groups (**Figures 5A–F**). The content of IL-17 in the control group was extremely low and could not be detected. In the LrB treatment groups, the contents of IL-1β, IL2, IL-6, IL-10 and IL-17 were significantly decreased (**Figures 5A–F**). We also applied qPCR to quantify the mRNA of IL-1β and IL-6 in joint cartilage of rats. Similar with ELISA test in serum, the IL-1β and IL-6 mRNA were significantly increased in CIA model group and decreased in LrB treatment group (**Figures 5G, H**). It is also interesting to explore whether the LrB treatment have effects on macrophages. We conducted qPCR to quantify the marker genes of macrophages and found that the iNOS, CD86 and CD206 mRNA were significantly higher in CIA model group while lower in LrB treatment group (**Figures 5I–K**). These results show that LrB can effectively reduce the content of IL-1β, IL-2, IL-6, IL-10 and IL-17 in the serum of RA rats, and inhibit the inflammatory reaction in CIA rats, in order to achieve the purpose of treatment.

### Effects of LrB on Kv1.3 expression in PBMCs and Treg cells in CIA rats

**Figure 6:**
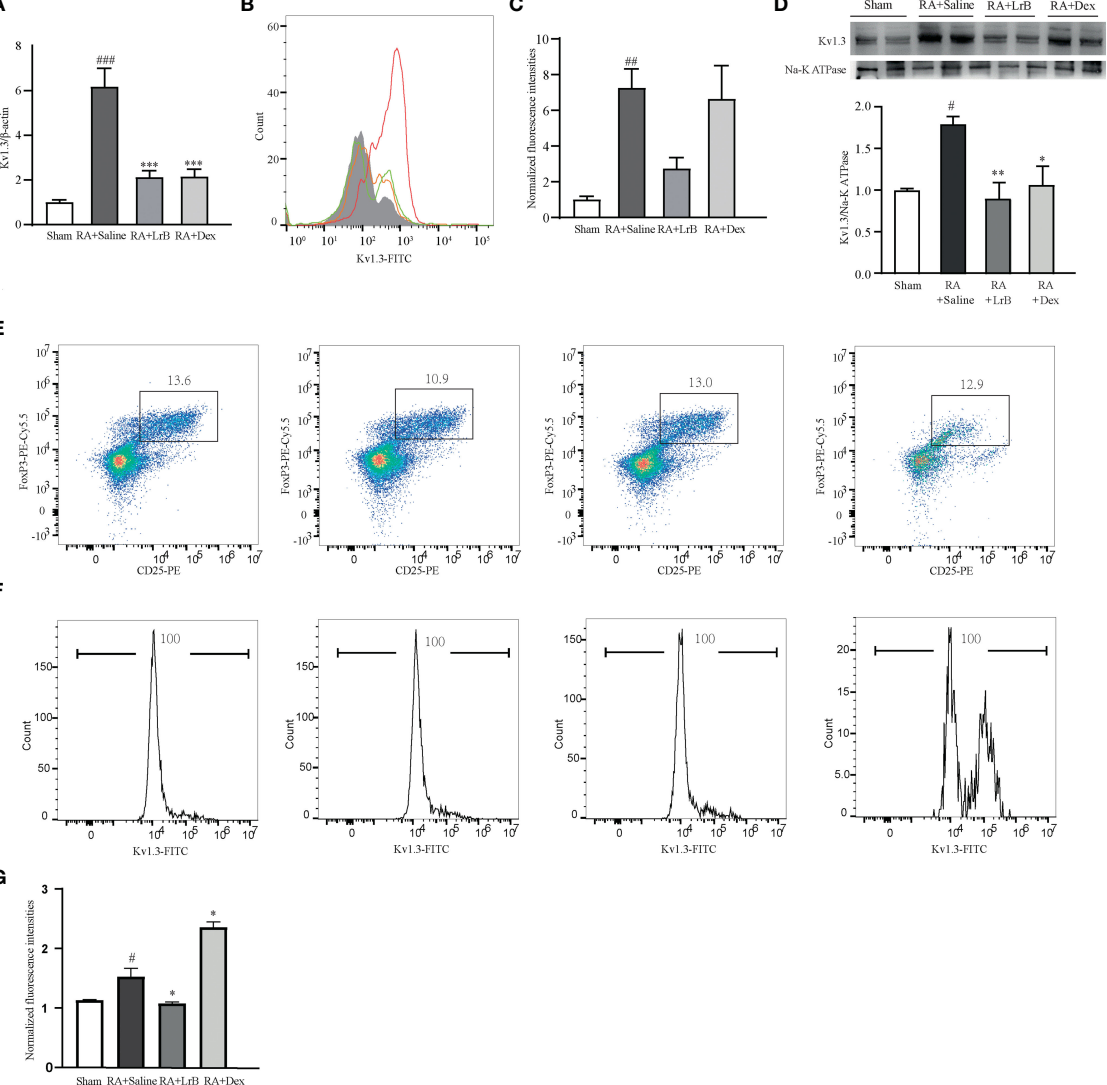
LrB decreased Kv1.3 expression in PBMCs and Treg cells in spleen. **(A)** Kv1.3 mRNA in PBMCs as shown by qPCR (n = 5). **(B, C)** The flow cytometry histogram **(B)** and average fluorescence intensity values **(C)** of Kv1.3 channel protein expressed on the membrane of PBMCs (n=4). **(D)** Kv1.3 expression was analyzed by western blot (n = 4). **(E)** Scatter diagram of CD25+FoxP3+ regulatory T-cell subtype (Treg cells) in CD4+ spleen cells of Sham and CIA rats with different treatments. **(F)** The flow cytometry histogram of Kv1.3-FITC fluorescence of Treg cells. **(G)** The statistics of FITC fluorescence of Treg cells (n=3). (Results are expressed as the mean ± SEM, and tested with One Way ANOVA. #, P ≤ 0.05, ##, P ≤ 0.01, compared to the Control group, * P ≤ 0.05, P ≤ 0.01, compared to the PHA stimulus group).

In view of the important roles of voltage-gated potassium channel Kv1.3 in the occurrence of RA, this study used qPCR technology, flow cytometry and Western Blot to determine whether LrB influenced the mRNA and protein levels of this molecule in PBMCs of CIA rats. The qPCR results indicated that Kv1.3 mRNA in PBMCs of the CIA model group was significantly higher than that in the control group (**Figure 6A**). In addition, LrB significantly down-regulated the mRNA level of Kv1.3 in PBMCs of CIA rats (**Figure 6A**). The results of flow cytometry and Western Blot also showed that Kv1.3 proteins in the PBMCs of CIA rats were significantly elevated (**Figures 6B–D**). LrB decreased the quantities of Kv1.3 but this decrease was not significant (**Figures 6B–D**). Kv1.3 play an important role in activation of T cells, especially the regulatory T (Treg) cells. We then labelled the rat spleen cells with anti-rat CD4-APC, CD25-PE, FoxP3-PE-Cy5.5 and Kv1.3-FITC antibodies and detected the CD4+CD25+FoxP3+ Treg cells using flow cytometry. Consistent with the changes in total PBMCs, the Kv1.3 fluorescence intensities in Treg cells of CIA model were significantly elevated and decreased to the Sham level in LrB treatment group (**Figures 6E–G**). We confirmed the changes in Kv1.3 protein and scrutinized the molecular mechanisms of immunosuppressive effects of LrB in Jurkat T cells and PBMCs.

### Effects of LrB on activated Jurkat T cells and PBMCs

**Figure 7:**
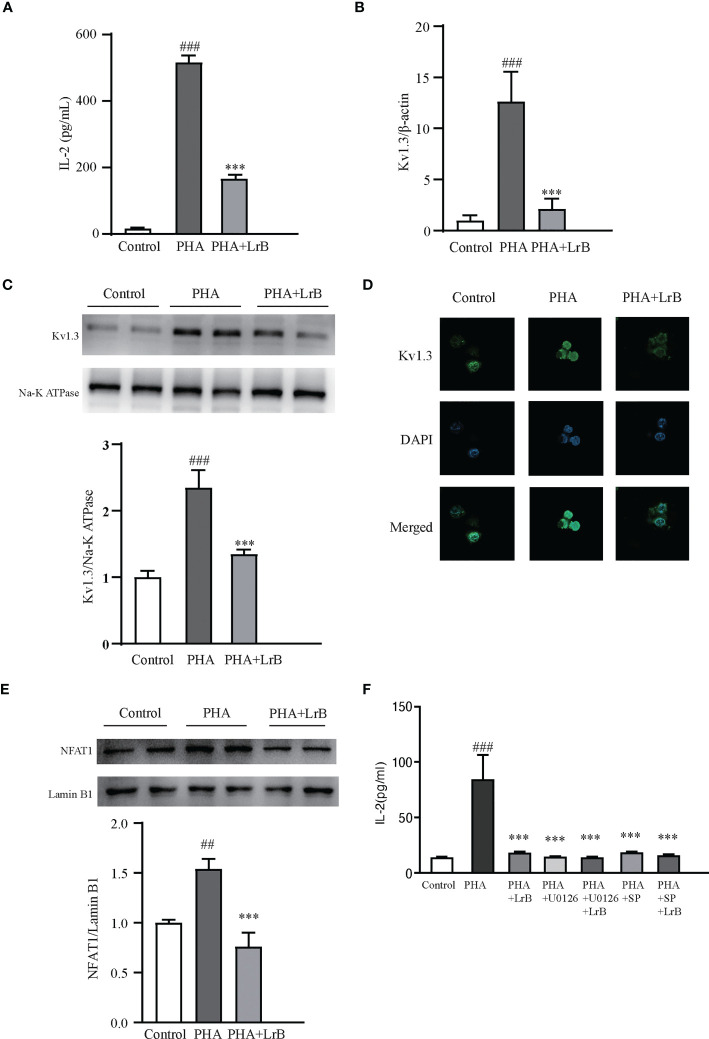
LrB decreased Kv1.3 expression and NFAT1 activation in Jurkat T cells. **(A)** The cytokine IL-2 released by Jurkat T cells detected by ELISA (n = 5). **(B)** Kv1.3 mRNA in PBMCs as shown by qPCR (n = 4). **(C)** Kv1.3 protein on the cell membrane was analyzed by western blot (n = 4). **(D)** Kv1.3 protein on the cell membrane was analyzed by immunofluorescence (n = 10). **(E)** NFAT1 in the nuclear region was analyzed by western blot (n = 6). **(F)** The cytokine IL-2 released by Jurkat T cells under the treatment of ERK inhibitor U0126, JNK inhibitor SP600125 and LrB (n = 3). (Results are expressed as the mean ± SEM, and tested with One Way ANOVA. ##, P ≤ 0.01, ###, P ≤ 0.001, compared to the Control group; * * * P ≤ 0.001, compared to the PHA stimulus group).

To investigate the molecular mechanisms of the immunosuppressive effects of LrB, we applied LrB in two cell models – the Jurkat T cell line and cultured PBMCs from rats. The stimulators PHA and ConA were separately added to the Jurkat T cell or PBMC culture medium after drug pre-treatment for 60 min, and the cells were placed in a 37 °C, 5% CO_2_ incubator for 24 h, and the samples were then collected. ELISA experiments were performed to detect the release of the inflammatory factor IL-2 by Jurkat T cells in the culture medium after activation by the stimulator. It was found that the cells activated by PHA significantly increased the expression and secretion of IL-2 (**Figure 7A**). LrB significantly decreased IL-2 release in the medium (**Figure 7A**). We also applied qPCR to detect the quantities of Kv1.3 mRNA and found it was consistent with the changes in IL-2 as shown by ELISA (**Figure 7B**). Western blot and immunofluorescence experiment of Kv1.3 also showed that Kv1.3 on the cell membrane of Jurkat T cells was significantly elevated by PHA stimulation and decreased by LrB treatment (**Figures 7C, D**).

It is reported that calcium ion (Ca^2+^) concentrations in activated T_EM_ cells can be significantly elevated (28). Our previous studies have shown that LrB inhibited the CRAC channel and decreased elevated Ca^2+^ concentrations in activated Jurkat T cells (18). The Ca^2+^ could bind to CaM and correspondingly activate the CaM-CaN-NFAT signaling pathway (29). Activated NFAT can then enter the nuclear region and regulate gene expression (29). It is of interest to test whether LrB can regulate the Ca^2+^ signaling pathway. We separated the proteins in the nuclear region and tested the quantities of NFAT1 by western blot. Our results showed that the quantity of NFAT1 in the nuclear region was significantly increased in the stimulator treated group (**Figure 7E**). LrB treatment significantly inhibited NFAT1 entering the nuclear region (**Figure 7E**). These results indicated that LrB could inhibit the activation of T cells by blocking the Ca^2+^ pathway and the activation of nuclear factor NFAT1. It was reported that LrB could also inhibit the activation of ERK and JNK (30). We applied p-ERK blocker U0126 and p-JNK blocker SP600125 in the Jurkat T cells and found that LrB did not further inhibit the IL-2 release under stimulation of PHA (**Figure 7F**). These results indicate that Kv1.3 might be the upstream molecule of ERK and JNK by which LrB inhibit the activation of lymphocytes.

**Figure 8:**
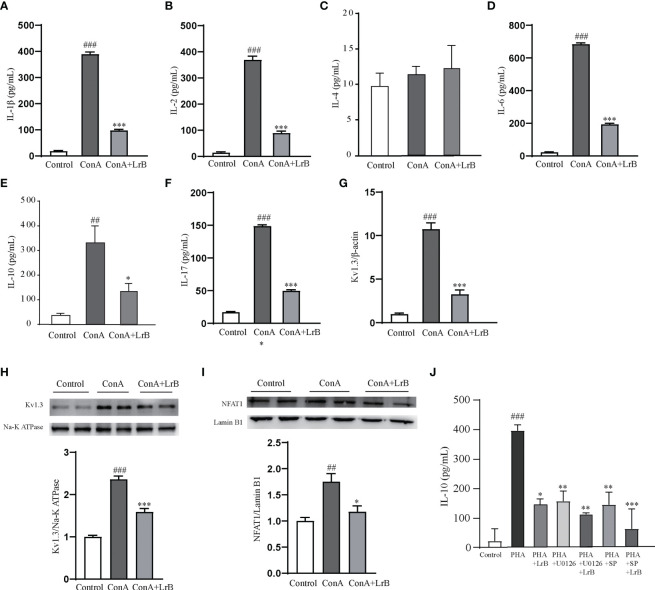
LrB decreased Kv1.3 expression and NFAT1 activation in PBMCs. **(A)** The concentration of IL-1β released by cultured PBMCs detected by ELISA (n=5). **(B)** The concentration of IL-2 released by cultured PBMCs (n = 5). **(C)** The concentration of IL-4 released by cultured PBMCs (n = 5). **(D)**. The concentration of IL-6 released by cultured PBMCs (n = 5). **(E)** The concentration of IL-10 released by cultured PBMCs (n=5). **(E)** Kv1.3 mRNA in PBMCs as shown by qPCR (n = 4). **(F)** The concentration of IL-17 released by cultured PBMCs (n=5). **(G)** Kv1.3 mRNA in PBMCs as shown by qPCR (n = 4). **(H)** Kv1.3 protein on the cell membrane was analyzed by western blot (n = 4). **(I)** NFAT1 in the nuclear region was analyzed by western blot (n=4). **(J)** The cytokine IL-10 released by PBMCs under the treatment of ERK inhibitor U0126, JNK inhibitor SP600125 and LrB (n = 3). (Results are expressed as the mean ± SEM, and tested with One Way ANOVA. ##, P ≤ 0.01, ###, P ≤ 0.001, compared to the Control group; * P ≤ 0.05, ** P ≤ 0.05, *** P ≤ 0.001, compared to the ConA stimulus group).

To confirm the effects and the signaling pathway in the T cell line, we also used ConA to stimulate cultured PBMCs extracted from rats. We observed that the released IL-1β, IL-2, IL-6, IL-10 and IL-17 in the ConA stimulated group were significantly increased (**Figures 8A–F**). In the LrB treatment groups, the levels of IL-1β, IL2, IL-6, IL-10 and IL-17 were significantly decreased (**Figures 8A–F**). In addition, the Kv1.3 mRNA and protein levels were increased in the stimulus treatment group and decreased by LrB accordingly (**Figures 8G, H**). The changes in NFAT1 in the nuclear region of PBMCs were similar to those in Jurkat T cells and LrB decreased the quantity of nuclear NFAT1 to the same level as that in the control group (**Figure 8I**). We also applied U0126 and SP600125 in the PBMCs and found that LrB did not further inhibit the IL-10 release under stimulation of ConA (**Figure 8J**). These results confirmed the phenomena in Jurkat T cells and prove the involvement of Kv1.3 and NFAT1 in regulating immune factor expression in lymphocytes. Further studies are needed to illustrate the function of Kv1.3 in immunosuppressive effects of LrB and the interactions between signalling pathways.

## Discussion

Chalcone and its derivatives are the products of aldol condensation of aromatic aldehydes and aromatic ketones (31). Chalcone exists widely as the parent compound in many natural plants, such as safflower, dragon’s blood, and is also the precursor compound of flavonoids in plants (32). The chalcone molecule can bind to different receptors due to its flexible structure, which may be the main reason for its extensive pharmacological activities. At present, many studies have shown that chalcone has anti-bacteria, anti-virus, anti-tumor and other pharmacological activities, and its mechanism of action is also more thorough (33, 34). However, there are relatively few reports on the immunosuppressive effect of chalcone, and even fewer on the mechanism of its immunosuppressive activity. LrB belongs to the dihydrochalcones and has a relatively simple structure and is easily synthesized and modified (1). Our previous research showed that naturally extracted LrB has immunosuppressive effects, which may be mediated by blocking the Kv1.3 channel (35). In order to further systematically explore the mechanism of LrB immunoregulation at the molecular, cellular and animal levels, LrB was chemically synthesized, and the structure identification was found to be completely correct (data not shown) (1). Electrophysiological experiments showed that the synthesized LrB efficiently blocked the Kv1.3 channel, providing a sufficient biological activity basis for pharmacological activity determination.

The CIA rat model can simulate RA in humans, and the therapeutic effects of LrB on RA were studied at the animal level (9). The animal experiment results showed that LrB had a good therapeutic effect in CIA rats, which significantly improved the clinical symptoms and tissue pathological changes in CIA rats. The infiltration of immune cells and the secretion of inflammatory factors could lead to the persistent synovitis in RA (8, 9). We tested the polarization of T cells in PBMCs and spleen cells and found that the proportion of CD4+ T cells were significantly increased in CIA model rats while decreased in LrB treatment group. The proportion of CD8+ T cells were significantly decreased in CIA model and increased in LrB treatment group. These results are consistent with previous studies that CD4+ T cells may promote the autoimmune responses in RA and CD8+ T cells may have a protective effects (36, 37). The cytokines play important roles in RA pathology and regulating the release of these cytokines may be effective treatment strategy for RA (8, 9). Except for the changes of T cell polarization, we found that LrB inhibited the release of activated T cell inflammatory cytokines IL-1β, IL-2, IL-6, IL-10 and IL-17, while there are no differences in IL-4. It is of interest to test the changes of other cytokines related to RA such as IFN-γ, IL-5, IL-12 and IL-13 (8). The activated macrophages could crosstalk with T cells to conduct the inflammatory effects in RA (38, 39). We thus applied qPCR to quantify the iNOS and CD86 which are marker genes for M1 macrophages, and CD206 which is marker gene for M2 macrophages (39, 40). Our results showed that iNOS, CD86 and CD206 increased significantly in CIA model and decreased after LrB treatment. The decrease of iNOS and CD86 indicated that LrB might suppress the activation of M1 macrophages. While CD206 is the marker gene for M2 macrophages which could release anti-inflammatory factors. We also quantified another marker gene for M2 macrophages - Arg1 with qPCR and found its mRNA in CIA model were significantly decreased and slightly elevated by LrB (data not shown). LrB might be able to activate M2 macrophages but down-regulate the expression of CD206 in these cells. We will explore the effects of LrB on polarization of M1/M2 macrophages in future.

We found that LrB not only directly blocked the Kv1.3 channel, but also down-regulated the expression of Kv1.3 in T lymphocytes of CIA rats. These two pharmacological effects may jointly mediate the effective inhibition by LrB on the autoimmune response of CIA rats. A variety of signal molecules and pathways are involved in the complex activation process of T cells. Among them, the T cell receptor (TCR) is the most basic signal molecule (41). A variety of chemical antigens, such as PHA and ConA can activate T lymphocytes by acting on the TCR, leading to T lymphocyte proliferation and the release of inflammatory cytokines (41). It can also increase the expression level of Kv1.3 channel on activated T lymphocyte membranes, which is consistent with the high expression of Kv1.3 channel protein on T_EM_ cell membranes in patients with autoimmune diseases (12, 42). In the animal study on RA treated with LrB, we found that LrB down-regulated Kv1.3 mRNA and protein in PBMCs of CIA rats. To investigate the changes of Kv1.3 channel in Treg cells, we further sorted the CD4+CD25+FoxP3+ cells in spleen and quantified the Kv1.3-FITC fluorescence. The Kv1.3 expression is higher in CIA model and was down-regulated in LrB treatment group. This pharmacological effect may supplement the important mechanism of the LrB mediated immunosuppressive effect by directly blocking Kv1.3 channels. It could be supported by the results of Dex group. Although Dex has been reported to greatly relieve RA symptoms, while it is not optimal because of the side effects (43). Our results showed that Dex significantly decreased the CD8+ cells in both PBMCs and spleen cells. And what surprise us is that Kv1.3 expression of Dex group is significantly elevated in PBMCs and Treg cells compared to Sham and even the CIA group. This indicates that Dex may activate rather than suppress the immune responses in RA and have deleterious effects on immune system. To further clarify whether LrB directly regulates the expression of Kv1.3 channels on T cell membranes, we used PHA activated Jurkat T cells and ConA activated rat PBMCs as cell models, and RT-PCR, Western Blot and immunofluorescence to detect the effects of LrB on the expression of Kv1.3 channels. The results showed that the mRNA and protein of Kv1.3 channels were both elevated, while treatment with LrB significantly decreased the expression levels of Kv1.3 channels, suggesting that LrB can directly inhibit the transcription of KCNA3 gene in T cells and the quantity of Kv1.3 channels on cell membranes.

When an antigen stimulates a T cell and binds to a TCR, downstream phospholipase C gamma (PLCγ) hydrolyzes PIP2 into DAG and inositol triphosphate (IP_3_) (44, 45). The newly synthesized IP_3_ binds to the IP_3_ receptor on the endoplasmic reticulum (ER) membrane and depletes calcium ion (Ca^2+^) stored in the ER pool (44, 45). Another ER membrane protein, stromal interaction molecule (STIM), can sense Ca^2+^ depletion in the ER and change its own conformation to contact and subsequently open the Orai Ca^2+^ channel on the plasma membrane (46). This process mediates Ca^2+^ influx and ultimately activates the downstream signaling pathways such as CaM-CaN-NFAT1 to produce inflammatory cytokines (13, 47). During these processes, the STIM1/Orai1 complex is the “major player” in triggering immune responses in T cells, while other ion channels, such as the potassium ion (K^+^) channel, can modulate calcium signals by changing the membrane potential of T cells and providing a driving force for Ca^2+^ entry (28). Kv1.3 is a predominantly expressed K^+^ channel in T cells and regulates immune responses stimulated by antigens (15, 28). Pharmacological blockade of Kv1.3 showed a strong therapeutic effect on RA model animals, suggesting that Kv1.3 is a new target for the discovery of specific RA immunosuppressive drugs (16, 17). Our previous studies showed that LrB can inhibit both Kv1.3 and STIM1/Orai1 channels and suppress the Ca^2+^ influx in Jurkat T cells stimulated by PHA, thus inhibiting IL-2 release from Jurkat T cells (18, 35). These results show that LrB might have modifying effects on autoimmune diseases by blocking the Ca^2+^ signaling pathway. In this study, we applied LrB in CIA rats and observed that LrB alleviated the symptoms in CIA model rats and suppressed cytokine release. Further *in vitro* studies in lymphocytes showed that LrB also inhibited the downstream molecule of the Ca^2+^ signaling pathway - transcription factor NFAT1 entering the nucleus. Because previous study have reported that LrB could exert its immunosuppressive effects through inhibiting ERK and JNK signalling molecules (30). We applied the ERK inhibitor U0126 and JNK inhibitor SP600125 in stimulated Jurkat T cells and PBMCs and quantified the release of inflammatory factors IL-2 and IL-10 separately. We found that LrB did not further decrease these cytokine release after inhibiting the ERK or JNK. This may indicate that ERK and JNK play central role in the immune regulation effects of LrB. Kv1.3 channel may be the upstream molecule which activates ERK and JNK. Further studies are needed to illustrate the relationship between Kv1.3 and the downstream molecular signals and the crosstalk between these signalling molecules. We will use Kv1.3 knockout mice to induce the CIA model and investigate the function of Kv1.3 in RA and other autoimmune diseases. Our results partly revealed the molecular mechanisms of the immunosuppressive effects of LrB which could provide a basis for drug development based on LrB.

## Data availability statement

The original contributions presented in the study are included in the article/**Supplementary Material**. Further inquiries can be directed to the corresponding authors.

## Ethics statement

The animal study was reviewed and approved by Institutional Animal Care and Use Committees (IACUC).

## Author contributions

YaZ and QZ contributed equally to this work. Conceived and designed the experiments: BH, SY. Performed the experiments: YaZ, QZ, XZ, HY, YoZ, ZL, MX, QX, LZ, WS, HT, LC. Analyzed the data: BH, SY, YaZ and QZ. Wrote the paper: QZ, SY. All authors contributed to the article and approved the submitted version.
