# Supplementary material for: The immunosuppressive effects and mechanisms of loureirin B on collagen-induced arthritis in rats
Source: Front Immunol. 2023 Apr 24;14:1094649. doi: 10.3389/fimmu.2023.1094649 (PMC10165104; doi:10.3389/fimmu.2023.1094649)
Supplement: Supplementary file 1 [file DataSheet_1.pdf]

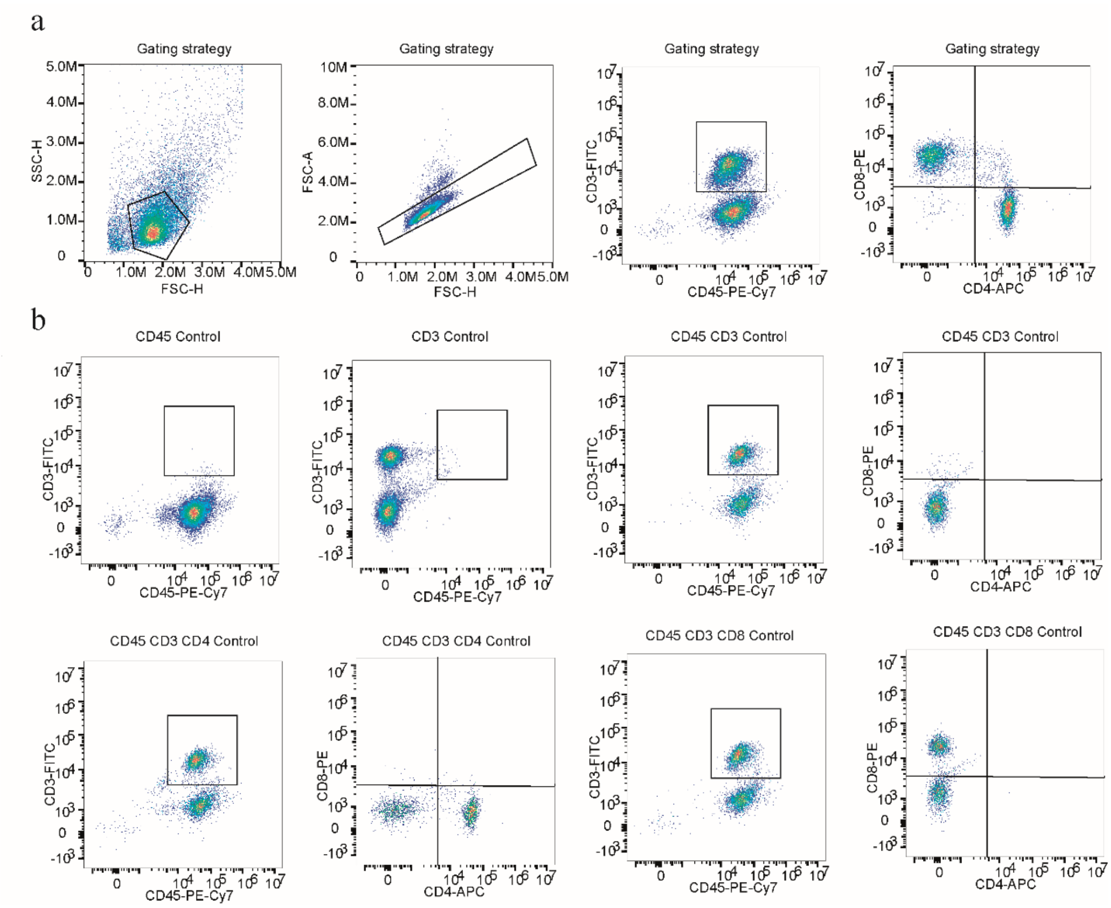

Supplementary figure1. Gating strategy and FMO control of T-cell subtype flow cytometry in spleen cells.

A. Gating strategy of T-cell subtype flow cytometry in spleen cells. B. FMO control of T-cell subtype flow cytometry in spleen cells.

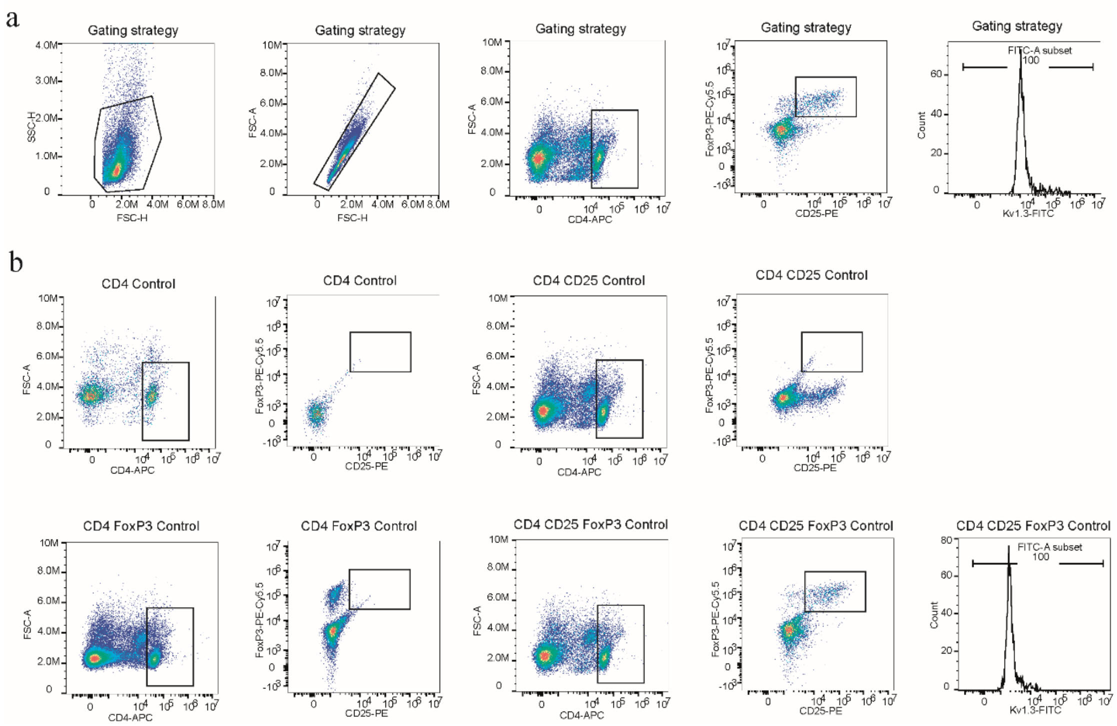

Supplementary figure2. Gating strategy and FMO control of regulatory T cell flow cytometry in spleen cells.

A. Gating strategy of regulatory T cell flow cytometry in spleen cells. B. FMO control of regulatory T cell flow cytometry in spleen cells.
